# The Effects of Soft Contact Lens Wear on The Tear Film and Meibomian Gland Drop-Out and Visibility

**DOI:** 10.3390/life12081177

**Published:** 2022-08-02

**Authors:** José Vicente García-Marqués, Cristian Talens-Estarelles, Santiago García-Lázaro, Alejandro Cerviño

**Affiliations:** Department of Optics and Optometry and Vision Sciences, University of Valencia, C/Dr. Moliner, 50, 46100 Valencia, Spain; jose.vicente.garcia@uv.es (J.V.G.-M.); cristian.talens@uv.es (C.T.-E.); santiago.garcia-lazaro@uv.es (S.G.-L.)

**Keywords:** contact lens, dry eye, meibomian glands, meibomian gland dysfunction, objective medical image analysis, ocular surface, tear film

## Abstract

As contact lens (CL) wear affects the ocular surface, this cross-sectional study aims to assess the effects of soft CL wear and its duration on the tear film and meibomian gland (MG) drop-out and visibility. Thirty non-CL wearers (22.5 ± 2.3 years) and twenty-four soft CL wearers (23.8 ± 2.2 years) participated in this study. The Keratograph 5M was used to assess the ocular surface. CL users were surveyed on years of CL wear and hours per week. MG visibility was assessed using a previously developed method based on analysing pixel intensity of meibographies. The CL group showed higher gland drop-out (*p* < 0.001) and lower gland visibility (*p* < 0.022). Gland drop-out was independently associated with CL wear (*p* = 0.006). When gland drop-out was excluded, the relative energy of pixel intensity values showed an independent association with CL wear (*p* = 0.005). Prolonged hours of CL wear were associated with higher dry eye symptoms and entropy of MGs (*p* < 0.029). A reduction in non-invasive keratograph break-up time was associated with using CLs for ≥8 years (*p* = 0.030). Overall, gland drop-out was higher and gland visibility lower in soft CL wearers. New gland visibility metrics might help to assess MGs in soft CL wearers quickly and objectively.

## 1. Introduction

The number of contact lens (CL) wearers worldwide is around 145 million, most of whom are soft CL wearers [1]. A CL splits the tear film into pre-lens tear film and post-lens tear film, which increases friction between the CL and the ocular surface. It has been estimated that approximately 30 to 50% of CL wearers report dry eye symptoms and discomfort, dryness being the most common complication [2,3]. Thus, dryness, discomfort, tearing, itching, hyperaemia and/or blurred vision are common signs and symptoms in CL wearers [4,5]. Consequently, dryness and lens discomfort could cause CL discontinuation and a reduction in the quality of life of CL wearers [3,6,7,8].

CL use has been acknowledged as a consistent risk factor for dry eye disease (DED) according to the Tear Film and Ocular Surface Society Dry Eye Workshop II (TFOS DEWS II) Epidemiology Report [1]. Previous work has reported more dry eye symptoms, tear film instability, thinner lipid layer, higher tear film osmolarity, lower tear meniscus and alterations in meibomian glands such as higher gland drop-out, thickening or poor gland expressibility in CL wearers compared to non-wearers [9,10,11,12,13].

The alteration of meibomian glands is a possible cause of CL-related dry eye [14]. Meibomian glands are sebaceous glands in the eyelids’ tarsal plate that secrete meibum [15,16]. Alterations in the function of meibomian glands can lead to meibomian gland dysfunction (MGD), a common and chronic disorder that causes alterations in the tear film, eye irritation, inflammation and ocular surface disease [15,17]. Some studies have assessed the effects of CL wear on meibomian glands and have reported lid margin abnormalities, gland obstruction, meibum abnormalities and morphological and functional alterations of meibomian glands with CL wear, consequently leading to an increase in ocular symptoms [7,14,18,19,20,21,22]. Nevertheless, other studies have found no association between CL wear and meibomian glands [23,24,25,26]. Likewise, the association between MGD and CL wear is still controversial and demonstrating a relationship between CL discomfort and MGD is challenging [7,18,26,27]. In this way, the effect of CLs on meibomian glands, the tear film and the ocular surface is still unclear, and some controversy remains. Moreover, it is not completely clear how the duration of CL wear impacts the ocular surface [1,3,27]. Overall, further studies are required to clarify this topic [1,28].

A new objective image-based method based on the visibility of meibomian glands has been recently developed [29]. Gland visibility has been proven to be used as a feature to aid in meibomian gland assessment. This method is based on analysing the grey intensity pixels of meibographies. As meibomian glands are lighter than the background, visibility is defined as the grade in which meibomian glands are seen, being low in individuals with high gland drop-out since glands have lost their visibility totally. Nevertheless, it has been demonstrated that meibomian gland visibility is not the same as meibomian gland drop-out since the authors of a recent study found different gland visibilities in groups with similar gland drop-out [29]. Thus, not only are gland visibility metrics useful in detecting gland drop-out, but they also grade the level of gland visibility in individuals with similar gland drop-out.

Gland visibility metrics correlate significantly with gland expressibility, tear film stability, tear meniscus and bulbar redness [30]. Moreover, authors [30] also found that meibomian gland visibility metrics have a good diagnostic capability to diagnose MGD, higher than current diagnostic metrics such as meibomian gland drop-out. The present study goes one step further by analysing, for the first time, the effect of soft CLs on meibomian gland visibility. It is hypothesized that these new metrics could be used as an objective and complementary tool to assess the effect of CLs on meibomian glands.

Due to the effect of CLs on the tear film and the ocular surface [31], the relevance of the tear film in CL fitting [32] and the controversy found in the effect of CLs on meibomian glands [1,3,27], the present study aims to assess the effect of soft CL wear and duration on meibomian glands, the tear film and ocular surface parameters. Furthermore, it will be studied whether a new method based on the analysis of the visibility of meibomian glands is able to assess changes in glands between CL wearers and non-wearers.

## 2. Materials and Methods

Thirty healthy non-CL wearers ranging in age between 18 to 30 years (22.5 ± 2.3 years) and twenty-four healthy, long-term soft CL wearers, with a duration of CL wear of at least 3 years and ranging in age between 19 to 27 years (23.8 ± 2.2 years) participated in this cross-sectional, prospective, clinical study. Participants who had never worn CLs made up the control group. No exclusion was made based on the tear film or meibomian gland parameters to assess different ocular surface and tear film status. Participants with ocular surface complications in the last 6 months were excluded from the study [9]. CL wearers were instructed to remove their CLs the night before the study and to attend the visit without wearing them [6,9]. Only the right eye of each participant was assessed to avoid data bias [6,14]. The study was carried out following the tenets of the Declaration of Helsinki and was approved by the Ethics Committee of the University of Valencia. Written consent from each participant was obtained after a verbal explanation of the protocol, nature and possible consequences of the study.

### 2.1. Measurements

The ocular surface of participants was assessed using the Oculus Keratograph 5M (K5 M; Oculus GmbH, Wetzlar, Germany) by the same experienced examiner within a single visit. Examiner was masked as to the group each participant belonged to. Measurements were performed following the guidelines of the TFOS DEWS II Diagnostic Methodology Report to avoid the destabilization of the tear film, in the following order [33]: Ocular Surface Disease Index (OSDI), 5-item Dry Eye Questionnaire (DEQ-5), total bulbar redness, tear meniscus height (TMH), non-invasive keratograph break-up time (NIKBUT) and upper eyelid meibography. All measurements were performed at the same time of the day to avoid the influence of diurnal variations [34], and the temperature and humidity of the room were maintained at 24.4 ± 1.5 °C and 44.8 ± 4.3%, respectively.

CL users were surveyed on their wearing habits, including years of CL wear and average days per week and hours per day of CL wear. The hours of CL wear per week were calculated by multiplying the days per week and hours per day.

The software automatically assessed bulbar redness, which detects the bulbar conjunctiva and obtains the ratio between conjunctival vessels (red) and sclera (white) with an accuracy of 0.1 units. The total bulbar redness score ranges from 0.0 to 4.0 [35]. Tear meniscus was captured immediately post-blink in primary gaze and TMH was measured in the middle of the eyelid using digital calipers as the distance between the upper limit of the reflective zone and the lower eyelid margin [33].

The first NIKBUT of the tear film was also assessed three consecutive times and averaged. Measurements were spaced for 3 min to allow tear film stabilization between readings [33]. Moreover, upper eyelid meibography was obtained using non-contact infrared meibography. The percentage of meibomian glands’ loss was calculated using the Image J tool (Wayne Rasband, National Institutes of Health, Bethesda, MD, USA), which has been previously used to evaluate gland drop-out objectively [36]. Meibomian gland drop-out percentage was obtained as the ratio between gland loss area and eyelid area.

**Figure 1:**
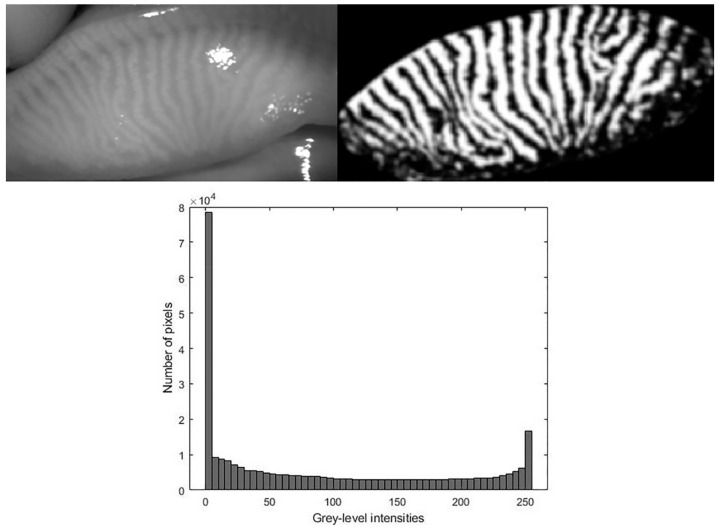
Meibography without image processing (**top left**), meibography after image processing (**top right**) and histogram of grey level intensity pixels of the processed meibography (**down**).

Meibomian gland visibility was assessed using a previously validated method (Figure 1) [29,30]. The mentioned method was developed using Matlab^©^ R2018a software (MathWorks, Natick, MA, USA) and different metrics based on the grey level intensity of pixel of meibographies were calculated: relative energy, energy, entropy, standard deviation (SD) irregularity, mean pixels intensity, SD pixels intensity, median pixels intensity, kurtosis and skewness. Further details on these parameters and their rationale can be found in previous reports [29,30]. Examiner was masked to the study group allocation of meibographies during analysis.

### 2.2. Statistical Analysis

Statistical analysis was performed using SPSS v26.0 for Windows (IBM Corp, Armonk, NY, USA). Results are shown as the mean ± SD. Normality distribution for each group and the total sample were assessed through the Shapiro-Wilk test. Differences in ocular surface parameters between groups (CL wearers and controls) were assessed using the *t*-Test for independent samples or the Mann-Whitney U test, depending on sample distribution. Sex differences between groups were evaluated using the Chi-square test. Moreover, participants with a gland drop-out higher than one-third of the total meibomian gland area [37] were excluded from the analysis to prove whether differences in gland visibility metrics resulted from gland drop-out.

Furthermore, univariate logistic regression was initially performed to identify potential predictors of CL wear. Parameters with a *p*-value less than 0.15 were incorporated into the multivariate logistic regression analysis [38]. Collinearity assumption was checked among variables. If some variables achieved a *p*-value < 0.15 but did not follow the assumption of collinearity, the parameter with the lowest *p*-value was included in the multivariate logistic regression analysis.

Correlations between ocular surface signs and symptoms and hours of CL use per week, and years of CL use were analysed using Pearson or Rho Spearman correlations for the CL group.

CL group was also divided into two groups depending on the years of CL wear and the hours of CL use per week. The median value was used as the cut-off value to divide the sample into two groups. The cut-off value for years of CL wear was 8 years, and the cut-off value for hours of CL use per week was 60 h. Differences in ocular surface parameters between groups (high or low use of CLs) were assessed using the *t*-Test for independent samples or the Mann-Whitney U test, depending on sample distribution. Sex differences between groups were evaluated using the Chi-square test. Finally, binomial logistic regression was performed to assess the predictability of ocular surface parameters to the duration of CLs use. A *p*-value less than 0.05 was defined as statistically significant.

## 3. Results

Thirty non-CL wearers (21 females and 9 males) and twenty-four long-term soft CL wearers (14 females and 10 males) were assessed in the present study. The mean age was 22.5 ± 2.3 years (ranging from 18 to 30 years old) and 23.8 ± 2.2 years (ranging from 19 to 27 years old) for the control and CL groups, respectively. No statistically significant differences in age or sex were found between groups (*p* = 0.254 and *p* = 0.650, respectively).

### 3.1. Differences between CL and Non-CL Wearers

Table 1 shows the main results obtained for each group. The CL group showed statistically and clinically higher gland drop-out and lower values in gland visibility metrics, except for SD irregularity and SD of region of interest (ROI) grey pixels intensity. Nevertheless, alterations in meibomian glands had no impact on dry eye symptoms, TMH, bulbar redness and NIKBUT (*p* > 0.005).

### 3.2. Differences between Groups without Participants with High Gland Drop-Out

Twelve participants in the CL group and two in the control group had a gland drop-out higher than one-third. The Chi-square test revealed that the proportion of participants with high gland drop-out was higher in the CL group than in the control (*p* = 0.008). No statistically significant differences were found in age between groups (*p* = 0.241).

Table 2 shows the mean values for each group and the comparison between them after excluding participants with high gland drop-out. No statistically significant differences were found between groups in any parameter (*p* > 0.05), suggesting that differences in gland visibility between groups were due to the higher gland drop-out in CL wearers.

### 3.3. Binomial Logistic Regression

Table 3 shows the univariate and multivariate-adjusted logistic regression analysis and the odds ratios for CL use for each parameter. Univariate logistic regression identified the following parameters as potential predictors of CL wear: gland drop-out, relative energy, energy, entropy, SD irregularity, mean pixels intensity, median pixels intensity, kurtosis and skewness. The interaction between them in the multivariate logistic regression revealed that gland drop-out percentage was independently associated with using CLs (*p* = 0.006). When gland drop-out was excluded from the analysis (Table 4), relative energy showed an independent association with CL wear (*p* = 0.005).

### 3.4. Relationship between Ocular Surface Parameters and Duration of CL Wear

Table 5 shows the correlations between ocular surface parameters and hours of CL wear per week and years of CL wear, respectively. Statistically significant correlations were found between hours of CL wear and DEQ-5 score, TMH and entropy of grey pixels intensity. Likewise, years of CL wear were positively correlated with the entropy of grey pixels intensity and negatively with NIKBUT. No other significant correlation was found with other ocular surface parameters.

Participants were also classified depending on the years of CL wear (Table 6). Twelve participants (7 females and 5 males) were classified into the group of CL wear <60 h per week and twelve (7 females and 5 males) in the group of CL wear ≥60 h per week. The mean age was 23.6 ± 2.2 and 23.9 ± 2.4 years old for participants wearing CLs <60 h per week and ≥60 h per week, respectively (*p* = 0.720). Moreover, eleven participants (6 females and 5 males) were classified into the group of CL wear <8 years and thirteen (8 females and 5 males) in the group of CL wear ≥8 years. The mean age was 23.4 ± 2.4 and 24.0 ± 2.3 years old for participants wearing CLs <8 years and ≥8 years, respectively (*p* = 0.654). The Chi-square test did not reveal statistically significant differences in sex between groups for hours per week (*p* = 0.685) and years of CL wear (*p* = 0.625).

Participants wearing CLs for more hours per week showed higher DEQ-5 and entropy values, whilst participants wearing CLs for more years had higher OSDI and entropy and lower NIKBUT. No statistically significant differences were found for the rest of the parameters assessed.

Table 7 shows the univariate and multivariate-adjusted logistic regression analysis, along with the odds ratios of ≥60 h of CL use per week and ≥8 years of CL use. Univariate logistic regression identified the following parameters as potential predictors of ≥60 h of CL use per week: DEQ-5, gland drop-out percentage, relative energy, entropy, SD irregularity, mean pixels intensity, median pixels intensity, kurtosis and skewness. The interaction between them in the multivariate logistic regression revealed that DEQ-5 and entropy were independently associated with using CLs ≥60 h per week (*p* < 0.029).

Univariate logistic regression identified the following parameters as potential predictors of ≥8 years of CL use: OSDI, NIKBUT and entropy. The interaction between them in the multivariate logistic regression revealed that NIKBUT was independently associated with using CLs ≥8 years (*p* = 0.030).

## 4. Discussion

### 4.1. Ocular Surface and CL Wear

It is widely known that CLs disturb the tear film and impact the ocular surface homeostasis [5,39]. Thus, the prevalence of dry eye in CL wearers is high and causes ocular discomfort, which alters the quality of life of individuals [5,7,8,39,40,41]. In this way, CL-associated dry eye is caused by tear film thinning due to the CL, which increases tear film evaporation, ocular surface inflammation, tear film osmolarity and lens de-wetting [12,14,42,43,44].

Nevertheless, the association between MGD and CL wear is still controversial [7,9,12,18,27,45,46], and while some studies have found morphological and functional alterations in meibomian glands in CL wearers [7,9,14,18,19,20,21,22,25,47], others have not found an association between CL wear and meibomian gland alterations [1,3,23,24,25,45]. The inconclusive association between CL wear and the meibomian glands supports the need for further studies that assess meibomian glands in CL wearers. The causative mechanism has been suggested to be the frictional and mechanical irritation caused by the lens [9,47] or the aggregation of desquamated epithelial cells at the orifices of the glands [19,20].

Since diagnosing DED and MGD is challenging, new metrics need to be developed to assess the ocular surface in an objective and non-invasive manner [33]. To the authors’ knowledge, this is the first study that objectively assessed meibomian gland visibility in CL wearers as well as its relationship with the time of CL wear and other ocular surface parameters. In the present study, meibomian gland drop-out was higher in the CL group. These results align with previous research that found alterations in meibomian glands in CL wearers [6,9,14,18,27,28,34,48,49,50]. Moreover, as meibomian gland drop-out is linked with meibomian gland visibility [29,30], lower gland visibility was also found in CL wearers. When participants with a gland drop-out higher than one-third were excluded from the analysis, it was proved that the low gland visibility found in CL wearers was a consequence of the gland drop-out in these participants since no differences were found between groups.

Multivariate logistic regression revealed that gland drop-out percentage was independently associated with using CLs. Each additional percentage of gland drop-out increased the probability of being in the CL group by 1.2 times. As changes in gland visibility resulted from gland drop-out in CL wearers, gland drop-out was excluded to assess whether gland visibility metrics were independently associated with the use of CLs. When gland drop-out was excluded from the analysis, relative energy was independently associated with using CLs. In another work [45], authors found that a higher meiboscore was independently associated with being a CL wearer, but not gland atrophy percentage.

These results do not mean that gland drop-out is a more robust metric than gland visibility metrics, but that gland visibility does not change independently of gland drop-out in CL wearers. Nevertheless, not only are gland visibility metrics useful to detect gland drop-out, but they also measure the visibility of glands. According to previous research, gland visibility metrics differ from gland drop-out because gland visibility could achieve different values depending on the visibility of glands in participants with similar gland drop-out [29]. Moreover, meibomian gland visibility metrics have good sensitivity and specificity to diagnose MGD, having higher diagnostic capability than current diagnostic metrics such as meibomian gland drop-out [30]. Results of the present study suggest that new metrics based on the visibility of meibomian glands can detect changes in the meibomian glands of CL wearers. However, despite gland visibility being different from gland drop-out [29], the changes in gland visibility in the present study are attributed to gland drop-out. Thus, gland visibility does not change independently of gland drop-out in CL wearers. Despite this, gland visibility metrics can detect alterations in meibomian glands in CL wearers, and they might be used in the follow-up of meibomian glands in these individuals as a quick, patient-friendly and objective method.

Dry eye symptoms, TMH, bulbar redness and NIKBUT tended to be worse in CL wearers, but the difference was not statistically significant between groups. Therefore, in the present study, CL wear did not cause changes in these parameters. This is in opposition to the results found in previous studies, which found lower tear meniscus [21,22] and more dry eye symptoms [6,9,22,34], bulbar redness [18] and tear film instability [9,14,21,22,34,48] in CL wearers as compared to non-wearers. In agreement with the results found in the present study, Arita et al. [14], Iqbal et al. [51] and Machalińska et al. [18] did not find differences in tear volume between CL and non-CL wearers. Likewise, other authors did not find statistically significant differences between CL and non-CL wearers for OSDI [18], break-up time [6,18,45], TMH, bulbar redness and tear osmolarity [45]. Differences between studies might be caused by differences in the methodological procedure since measurements were taken only a few hours after CL removal in some studies, which could have caused changes in ocular surface parameters. Moreover, there are also differences in the minimum period of years of CL wear to include a subject in the CL group. In the present work, CL wearers used CLs for at least 3 years and were instructed to remove them the night before the examination, following the methodology of previous works [6,9,18,21,45].

### 4.2. Relationship between Ocular Surface Parameters and Duration of CL Wear

The effect of the duration of CL wear on meibomian glands and tear film also has some controversy [14,21,23,27]. In the present work, hours of CL wear per week was positively correlated with DEQ-5 and entropy; and negatively correlated with TMH. Thus, participants who used CLs for more hours per week had less TMH and higher dry eye symptoms and entropy in meibomian glands. Years of CL wear were also negatively correlated with NIKBUT and positively correlated with entropy. Entropy measures the randomness of the grey level distribution [52], which might be helpful for the analysis of parameters related to meibography arrangements, such as tortuosity of glands [29]. This might suggest that participants that used CL for more hours per week and more years had higher tortuosity in meibomian glands. Previous work [48] also found morphological irregularities in meibomian glands in symptomatic CL wearers.

The present study found no association between CL duration and gland drop-out and visibility. In agreement with the results of this study, other authors [21,50] found that alterations in meibomian glands are either long-term or take place in an early phase of CL wear. Thus, Alghamdi et al. [21] found changes in meibomian glands during the first two years of CL wear, but prolonged exposure to CL beyond this point was not associated with further changes in meibomian glands. Some adaptive mechanisms in the eyelid tissues might explain this [21]. Therefore, this might explain why this study found no differences in gland drop-out. As CL wearers in the present study reported using CLs for at least three years, meibomian gland drop-out might have occurred in the first years of CL wear, being stable after that early phase of CL use. On the other hand, these results suggest that gland tortuosity might increase with CL duration since the entropy of glands increased.

This is in opposition to the results of some authors that found that the function and morphology of meibomian glands are related to the duration of CL wear [6,9,14,18,22]. For instance, Arita et al. [14] found a significant positive correlation between meiboscore and the duration of CL wear. Nevertheless, both soft and rigid CL wearers were included in the study and participants with at least one year of CL wear were included, which is lower than the minimum years of lens wear in the present study. In another work [9], authors claimed that the meibomian glands of CL wearers deteriorate after six years of wear but remain stable thereafter. Harbiyeli et al. [6] also found that the hours of soft CL use were associated with the loss of meibomian glands in multivariate analysis. However, the duration of CL use was less than five years in more than half of the sample and they mixed soft and rigid CL wearers, the latter being more likely to experience more pronounced MGD. Therefore, this study might also confirm that changes in meibomian gland drop-out occur in the first years of CL use, which agrees with the results found in the present work. Moreover, the present study adds that the duration of CL wear might be related to gland tortuosity.

In the present work, the median pixel intensity, kurtosis and skewness were almost statistically significant and tended to be worse in the group with ≥60 h of CL use per week. Thus, despite CL wearers having higher gland drop-out and lower gland visibility, no relationship was found between the time of CL use and gland drop-out and gland visibility, except for entropy. This suggests that no changes in gland drop-out and gland visibility occur from three years of CL use. Nevertheless, gland drop-out and kurtosis were identified as potential predictors of using CLs for ≥60 h per week. Multivariate logistic regression revealed that DEQ-5 score and entropy were independently associated with using CLs for ≥60 h per week. Each additional score in DEQ-5 and entropy increased the probability of being in the group with high CLs use by 1.33 and 1.30 times, respectively.

Moreover, OSDI score, NIKBUT and entropy were identified as potential predictors of being a CL wearer for ≥8 years. Previous work [9] also found a higher OSDI score in individuals wearing CLs for more than seven years and a lower break-up time in those wearing CLs for more than seven years and between three and seven years in comparison with a control group. Furthermore, tear film break-up time also worsened after three years of wear but remained stable after six years. In the present study, multivariate logistic regression revealed that NIKBUT was independently associated with being a CL wearer for ≥8 years. Every second decrease in NIKBUT increased the probability of being in the group with high use of CLs by 1.34 times.

The present study had some limitations to consider. First, small sample size was used to study the effect of the duration of contact lens wear on meibomian glands. However, it is important to bear in mind that this is the first study assessing the effect of soft contact lens wear on these new metrics based on the assessment of meibomian gland visibility. Furthermore, participants were instructed to remove their CLs the night before the study and to attend the visit without wearing them [6,9,18]. This could have caused no statistically significant differences between groups in some parameters such as NIKBUT, TMH or bulbar redness. Moreover, studies with different methodological procedures are not directly comparable. In addition, the method to measure gland visibility is semiautomatic and the clinician still has to eliminate reflexes and manually delineate the area of the glands. Nevertheless, the method is not instrument-specific, and despite being semiautomatic, the repeatability was found to be acceptable [29]. Furthermore, only the upper eyelids were assessed since capturing a uniformly focused image of the tarsal plate was easier. Arita et al. [14] found that the meiboscore in the upper eyelid was higher than in the lower eyelid in CL wearers. This might be because the upper eyelid becomes more irritated as it makes more extended movements during blinking [14]. In another study [9], the authors found that CLs influence upper eyelids in the early years of CL wear, but both eyelids were equally affected after three years. Therefore, in the present study, no differences between upper and lower eyelids are expected since all participants were CL wearers for at least three years. The present work was limited to soft CL wearers and differences with other CL designs or materials were not assessed, which might be studied in future studies. Finally, this work did not assess meibomian gland expressibility and lid margin abnormalities. However, gland visibility metrics have been reported to be correlated with the gland expressibility [30].

## 5. Conclusions

Overall, the present work adds valuable information regarding the effect of soft CL wear on the tear film, meibomian gland drop-out and meibomian gland visibility. Gland drop-out was higher and gland visibility lower in long-term soft CL wearers in compared to controls. Nevertheless, gland visibility changed in CL wearers due to gland drop-out. Prolonged hours of CL wear are associated with higher dry eye symptoms and entropy of meibomian glands, which might be a measurement of gland tortuosity; whilst more years of CL use is related to a reduction in NIKBUT. Therefore, CL wear might aggravate dryness and the alterations in meibomian glands that take place at advanced ages even more. Clinicians should assess the ocular surface in CL wearers and educate them to use CLs properly to maintain healthy meibomian glands and ocular surface. New gland visibility metrics might help to assess the follow-up of meibomian glands in soft CL wearers in a quickly and objectively. Further studies are needed to confirm these preliminary results and to assess the effect of different soft CLs with other materials and designs on meibomian glands depending on DED diagnosis, in different age groups, with larger sample sizes and with longer follow-up periods. A prospective, longitudinal study is required to understand associations between the alterations of meibomian glands and CL wear.

## Figures and Tables

**Table 1:** Mean values for each group and comparison between them.

Metric	Control Group (Mean ± SD) (30 Subjects)	Contact Lens Group (Mean ± SD) (24 Subjects)	Significance Level (*p*-Value)
OSDI	14.9 ± 12.6	18.3 ± 16.7	0.978 ‡
DEQ-5	6.2 ± 5.1	7.1 ± 4.4	0.687 †
TMH (mm)	0.22 ± 0.05	0.20 ± 0.07	0.292 †
Bulbar redness	0.50 ± 0.24	0.60 ± 0.26	0.381 ‡
NIKBUT (seconds)	14.8 ± 8.0	16.9 ± 8.5	0.526 ‡
Drop-out percentage (%)	25.5 ± 6.8	37.7 ± 11.4	<0.001 †*
Relative energy	0.38 ± 0.06	0.30 ± 0.09	<0.001 †*
Energy	245.1 ± 4.9	238.6 ± 7.3	0.012 †*
Entropy	3.8 × 10^−5^ ± 9.2 × 10^−6^	4.6 × 10^−5^ ± 1.0 × 10^−5^	0.022 ‡*
SD Irregularity	0.28 ± 0.07	0.23 ± 0.08	0.069 †
Mean ROI pixels intensity	109.4 ± 11.8	95.7 ± 17.2	0.004 †*
SD ROI pixels intensity	76.4 ± 5.7	74.3 ± 8.4	0.283 †
Median ROI pixels intensity	92.4 ± 17.8	72.2 ± 21.4	<0.001 †*
Kurtosis	0.0103 ± 0.0006	0.0116 ± 0.019	0.001 †*
Skewness	0.103 ± 0.004	0.109 ± 0.009	0.001 †*

Where: DEQ-5 = 5-item Dry Eye Questionnaire; NIKBUT = non-invasive keratograph break-up time; OSDI = Ocular Surface Disease Index; ROI = region of interest; SD = standard deviation; TMH = tear meniscus height; * = statistically significant;† *t*-Test.; ‡ Mann-Whitney U test.

**Table 2:** Mean values for the control and the CL groups and comparison between them after excluding participants with a gland-out higher than one-third of the total meibomian gland area.

Metric	Control Group (Mean ± SD) (30 Subjects)	Contact Lens Group (Mean ± SD) (24 Subjects)	Significance Level (*p*-Value)
Age (years)	22.5 ± 2.4	23.7 ± 3.1	0.241 †
OSDI	14.9 ± 12.6	10.0 ± 9.3	0.398 ‡
DEQ-5	6.2 ± 5.1	4.7 ± 3.6	0.602 †
TMH (mm)	0.22 ± 0.05	0.18 ± 0.06	0.109 †
Bulbar redness	0.50 ± 0.23	0.53 ± 0.0.22	0.752 †
NIKBUT (seconds)	14.3 ± 8.1	13.9 ± 9.3	0.887 ‡
Drop-out percentage (%)	24.5 ± 6.1	28.8 ± 4.9	0.124 †
Relative energy	0.39 ± 0.60	0.36 ± 0.67	0.313 †
Energy	245.0 ± 5.1	243.0 ± 9.7	0.482†
Entropy	4.5 × 10^−5^ ± 9.6 × 10^−6^	4.3 × 10^−5^ ± 8.2 × 10^−6^	0.606 †
SD Irregularity	0.28 ± 0.08	0.27 ± 0.08	0.670 †
Mean ROI pixels intensity	109.8 ± 12.2	104.6 ± 13.7	0.371 †
SD ROI pixels intensity	76.1 ± 5.8	76.6 ± 6.5	0.874 †
Median ROI pixels intensity	93.7 ± 17.9	85.0 ± 19.8	0.308 †
Kurtosis	0.0102 ± 0.0006	0.0107 ± 0.0012	0.216 †
Skewness	0.103 ± 0.003	0.105 ± 0.005	0.249 †

Where: DEQ-5 = 5-item Dry Eye Questionnaire; NIKBUT = non-invasive keratograph break-up time; OSDI = Ocular Surface Disease Index; ROI = region of interest; SD = standard deviation; TMH = tear meniscus height; † *t*-Test; ‡ Mann-Whitney U test.

**Table 3:** Univariate and multivariate logistic regressions and odds ratios of CL wear for demographic and clinical characteristics.

Characteristic	Univariate Logistic Regression				Multivariate Logistic Regression			
	Odd Ratio	Lower CI	Upper CI	*p*-Value	Odd Ratio	Lower CI	Upper CI	*p*-Value
Age	1.054	0.995	1.029	0.284				
Sex	1.050	0.164	6.724	0.959				
OSDI	1.018	0.960	1.080	0.553				
DEQ-5	1.043	0.860	1.264	0.669				
TMH (mm)	0.001	0.000	326.650	0.284				
Bulbar redness	4.779	0.274	83.313	0.283				
NIKBUT (seconds)	1.032	0.945	1.127	0.486				
Drop-out percentage (%)	1.203	1.051	1.376	0.007 *	1.207	1.053	1.382	0.006 *
Relative energy	0.000	0.000	0.029	0.013 *	0.000	0.000	5.235	0.521
Energy	0.829	0.823	1.049	0.029 *				
Entropy	1.182	1.094	2.084	0.015 *	1.152	1.052	1.810	0.282
SD Irregularity	0.000	0.000	3.321	0.081				
Mean ROI pixels intensity	0.927	0.869	0.989	0.022 *				
SD ROI pixels intensity	0.954	0.860	1.058	0.375				
Median ROI pixels intensity	0.944	0.902	0.988	0.014 *				
Kurtosis	1.782	1.098	8.698	0.022 *				
Skewness	1.487	1.424	1.553	0.024 *				

Where: DEQ-5 = 5-item Dry Eye Questionnaire; NIKBUT = non-invasive keratograph break-up time; OSDI = Ocular Surface Disease Index; ROI = region of interest; SD = standard deviation; TMH = tear meniscus height; CI = 95% confidence interval. * = statistically significant values.

**Table 4:** Multivariate logistic regressions and odds ratios of CL use after excluding gland drop-out percentage.

	Multivariate Logistic Regression			
	Odd Ratio	Lower CI	Upper CI	*p*-Value
Relative energy	0.000	0.000	0.021	0.005 *
Entropy	1.224	1.119	1.523	0.213

Where: CI = 95% confidence interval. * = statistically significant values.

**Table 5:** Correlations between ocular surface parameters and hours of CL wear per week and years of CL wear.

	Metric	Correlation Coefficient (r)	Significance Level (*p*-Value)
Hours of contact lens use per week (24 subjects)	OSDI	0.292	0.111 ‡
	DEQ-5	0.387	0.031 †*
	TMH (mm)	−0.357	0.049 ‡*
	Bulbar redness	0.182	0.328 †
	NIKBUT (seconds)	−0.254	0.169 ‡
	Drop-out percentage (%)	0.053	0.778 †
	Relative energy	−0.203	0.274 †
	Energy	−0.176	0.345 ‡
	Entropy	0.436	0.014 ‡*
	SD Irregularity	−0.213	0.251 †
	Mean ROI pixels intensity	−0.235	0.203 †
	SD ROI pixels intensity	−0.272	0.138 †
	Median ROI pixels intensity	−0.212	0.253 †
	Kurtosis	0.254	0.168 †
	Skewness	0.248	0.179 †
Years wearing contact lenses (24 subjects)	OSDI	0.269	0.144 ‡
	DEQ-5	0.089	0.635 ‡
	TMH (mm)	−0.069	0.710 ‡
	Bulbar redness	0.038	0.838 ‡
	NIKBUT (seconds)	−0.355	0.045 ‡*
	Drop-out percentage (%)	0.037	0.842 ‡
	Relative energy	−0.073	0.697 ‡
	Energy	−0.210	0.258 ‡
	Entropy	0.382	0.034 ‡*
	SD Irregularity	−0.116	0.533 ‡
	Mean ROI pixels intensity	−0.120	0.521 ‡
	SD ROI pixels intensity	−0.161	0.387 ‡
	Median ROI pixels intensity	−0.008	0.965 ‡
	Kurtosis	0.016	0.934 ‡
	Skewness	0.032	0.865 ‡

Where: DEQ-5 = 5-item Dry Eye Questionnaire; NIKBUT = non-invasive keratograph break-up time; OSDI = Ocular Surface Disease Index; ROI = region of interest; SD = standard deviation; TMH = tear meniscus height; * = statistically significant; † Pearson coefficient; ‡ Spearman coefficient.

**Table 6:** Comparison of ocular surface parameters depending on hours of CL wear per week and years of CL wear.

Metric	<60 h per Week (Mean ± SD) (12 Subjects)	≥60 h per Week (Mean ± SD) (12 Subjects)	Significance Level (*p*-Value)
Weekly hours wearing contact lenses	29.1 ± 17.2	77.2 ± 13.0	<0.001 ‡*
OSDI	12.9 ± 10.8	19.9 ± 21.0	0.423 ‡
DEQ-5	5.3 ± 3.5	8.3 ± 3.6	0.028 †*
TMH (mm)	0.22 ± 0.06	0.18 ± 0.06	0.167 †
Bulbar redness	0.62 ± 0.27	0.65 ± 0.34	0.758 †
NIKBUT (seconds)	16.6 ± 5.5	15.0 ± 5.3	0.379 ‡
Drop-out percentage (%)	35.1 ± 9.4	37.4 ± 10.2	0.095 †
Relative energy	0.34 ± 0.06	0.30 ± 0.08	0.105 †
Energy	239.0 ± 2.9	237.5 ± 5.1	0.626 ‡
Entropy	3.8 × 10^−5^ ± 6.0 × 10^−6^	4.6 × 10^−5^ ± 1.2 × 10^−5^	0.027 ‡*
SD Irregularity	0.23 ± 0.08	0.21 ± 0.09	0.142 †
Mean ROI pixels intensity	99.0 ± 11.7	90.9 ± 15.3	0.108 †
SD ROI pixels intensity	74.9 ± 6.0	74.5 ± 5.3	0.251 †
Median ROI pixels intensity	78.7 ± 16.7	70.7 ± 23.1	0.082 †
Kurtosis	0.0113 ± 0.0018	0.0116 ± 0.0019	0.051 †
Skewness	0.106 ± 0.004	0.109 ± 0.008	0.057 †
**Metric**	**<8 Years of Contact Lens Wear (Mean ± SD) (12 Subjects)**	**≥8 Years of Contact Lens Wear (Mean ± SD) (12 Subjects)**	**Significance Level (*p*-Value)**
Years wearing contact lenses	5.0 ± 1.0	11.9 ± 2.5	<0.001 †*
OSDI	18.6 ± 17.9	26.3 ± 13.8	0.048 ‡*
DEQ-5	7.6 ± 2.8	7.9 ± 4.5	0.852 †
TMH (mm)	0.20 ± 0.09	0.19 ± 0.07	0.544 ‡
Bulbar redness	0.62 ± 0.27	0.65 ± 0.34	0.732 †
NIKBUT (seconds)	17.0 ± 6.1	10.1 ± 2.4	0.008 †*
Drop-out percentage (%)	36.7 ± 10.7	37.6 ± 10.8	0.729 †
Relative energy	0.30 ± 0.08	0.29 ± 0.07	0.728 †
Energy	238.5 ± 3.1	238.0 ± 5.3	0.570 ‡
Entropy	3.9 × 10^−5^ ± 1.0 × 10^−5^	4.6 × 10^−5^ ± 1.0 × 10^−5^	0.039 ‡*
SD Irregularity	0.23 ± 0.09	0.21 ± 0.09	0.523 †
Mean ROI pixels intensity	96.3 ± 17.7	94.1 ± 16.7	0.679 †
SD ROI pixels intensity	76.3 ± 7.7	73.2 ± 6.9	0.450 †
Median ROI pixels intensity	75.3 ± 23.2	70.6 ± 19.3	0.832 †
Kurtosis	0.0116 ± 0.0018	0.0116 ± 0.0019	0.940 †
Skewness	0.109 ± 0.008	0.109 ± 0.009	0.897 †

Where: DEQ-5 = 5-item Dry Eye Questionnaire; NIKBUT = non-invasive keratograph break-up time; OSDI = Ocular Surface Disease Index; ROI = region of interest; SD = standard deviation; TMH = tear meniscus height; * = statistically significant; † *t*-Test; ‡ Mann-Whitney U test.

**Table 7:** Univariate and multivariate logistic regressions and odds ratios of CL use ≥60 h per week and CL use ≥8 years for demographic and clinical characteristics.

CL Use ≥60 h								
Characteristic	Univariate Logistic Regression				Multivariate Logistic Regression			
	Odd Ratio	Lower CI	Upper CI	*p*-Value	Odd Ratio	Lower CI	Upper CI	*p*-Value
Age	1.074	0.894	1.292	0.446				
Sex	1.818	0.350	9.455	0.477				
OSDI	1.017	0.922	1.098	0.197				
DEQ-5	1.027	1.012	1.595	0.039 *	1.330	1.028	1.718	0.029 *
TMH (mm)	0.001	0.000	28.250	0.176				
Bulbar redness	1.477	0.136	16.130	0.749				
NIKBUT (seconds)	0.944	0.822	1.085	0.418				
Drop-out percentage (%)	1.100	0.980	1.234	0.105	1.044	0.789	1.382	0.762
Relative energy	0.000	0.000	8.264	0.111				
Energy	0.905	0.752	1.091	0.296				
Entropy	2.837	1.390	57.890	0.044 *	1.298	1.194	2.857	0.042 *
SD Irregularity	0.000	0.000	9.524	0.105				
Mean ROI pixels intensity	0.955	0.902	1.011	0.113				
SD ROI pixels intensity	0.888	0.728	1.085	0.244				
Median ROI pixels intensity	0.966	0.929	1.006	0.092				
Kurtosis	1.182	1.088	6.698	0.064	1.222	0.935	1.423	0.188
Skewness	1.492	1.399	6.501	0.068				
**CL Use ≥8 Years**								
**Characteristic**	**Univariate Logistic Regression**				**Multivariate Logistic Regression**			
	**Odd Ratio**	**Lower CI**	**Upper CI**	***p*-Value**	**Odd Ratio**	**Lower CI**	**Upper CI**	***p*-Value**
Age	1.135	0.934	1.379	0.204				
Sex	1.152	0.958	8.480	0.421				
OSDI	1.223	0.976	1.973	0.123	1.012	0.959	1.068	0.662
DEQ-5	1.023	0.846	1.236	0.818				
TMH (mm)	0.027	0.000	442.450	0.466				
Bulbar redness	1.546	0.142	16.949	0.721				
NIKBUT (seconds)	0.766	0.607	0.967	0.025 *	0.745	0.570	0.972	0.030 *
Drop-out percentage (%)	1.019	0.921	1.128	0.718				
Relative energy	0.152	0.000	4094.118	0.718				
Energy	0.972	0.817	1.156	0.748				
Entropy	1.481	1.290	3.210	0.089	1.431	1.105	1.673	0.216
SD Irregularity	0.059	0.000	260.172	0.508				
Mean ROI pixels intensity	0.989	0.939	1.041	0.668				
SD ROI pixels intensity	0.926	0.763	1.124	0.436				
Median ROI pixels intensity	0.996	0.962	1.031	0.825				
Kurtosis	1.186	1.018	1.526	0.937				
Skewness	1.158	1.025	1.516	0.892				

Where: DEQ-5 = 5-item Dry Eye Questionnaire; NIKBUT = non-invasive keratograph break-up time; OSDI = Ocular Surface Disease Index; ROI = region of interest; SD = standard deviation; TMH = tear meniscus height; CI = 95% confidence interval. * = Statistically significant values.
